# Comparative Genomic Analysis of Globally Dominant ST131 Clone with Other Epidemiologically Successful Extraintestinal Pathogenic *Escherichia coli* (ExPEC) Lineages

**DOI:** 10.1128/mBio.01596-17

**Published:** 2017-10-24

**Authors:** Sabiha Shaik, Amit Ranjan, Sumeet K. Tiwari, Arif Hussain, Nishant Nandanwar, Narender Kumar, Savita Jadhav, Torsten Semmler, Ramani Baddam, Mohammed Aminul Islam, Munirul Alam, Lothar H. Wieler, Haruo Watanabe, Niyaz Ahmed

**Affiliations:** a Pathogen Biology Laboratory, Department of Biotechnology and Bioinformatics, University of Hyderabad, Gachibowli, Hyderabad, India; b Department of Microbiology, Dr. D. Y. Patil Medical College, Hospital and Research Centre (Dr. D. Y. Patil Vidyapeeth), Pimpri, Pune, India; c Centre for Infection Medicine, Institute of Microbiology and Epizootics, Freie Universität Berlin, Berlin, Germany; d Robert Koch Institute, Berlin, Germany; e International Center for Diarrheal Disease Research Bangladesh (icddr,b), Mohakhali, Dhaka, Bangladesh; f National Institute of Infectious Diseases, Tokyo, Japan; GSK Vaccines

**Keywords:** bacterial evolution, *Escherichia coli*, genomics, ST131 lineage, molecular epidemiology

## Abstract

*Escherichia coli* sequence type 131 (ST131), a pandemic clone responsible for the high incidence of extraintestinal pathogenic *E. coli* (ExPEC) infections, has been known widely for its contribution to the worldwide dissemination of multidrug resistance. Although other ExPEC-associated and extended-spectrum-β-lactamase (ESBL)-producing *E. coli* clones, such as ST38, ST405, and ST648 have been studied widely, no comparative genomic data with respect to other genotypes exist for ST131. In this study, comparative genomic analysis was performed for 99 ST131 *E. coli* strains with 40 genomes from three other STs, including ST38 (*n =* 12), ST405 (*n =* 10), and ST648 (*n =* 18), and functional studies were performed on five in-house strains corresponding to the four STs. Phylogenomic analysis results from this study corroborated with the sequence type-specific clonality. Results from the genome-wide resistance profiling confirmed that all strains were inherently multidrug resistant. ST131 genomes showed unique virulence profiles, and analysis of mobile genetic elements and their associated methyltransferases (MTases) has revealed that several of them were missing from the majority of the non-ST131 strains. Despite the fact that non-ST131 strains lacked few essential genes belonging to the serum resistome, the in-house strains representing all four STs demonstrated similar resistance levels to serum antibactericidal activity. Core genome analysis data revealed that non-ST131 strains usually lacked several ST131-defined genomic coordinates, and a significant number of genes were missing from the core of the ST131 genomes. Data from this study reinforce adaptive diversification of *E. coli* strains belonging to the ST131 lineage and provide new insights into the molecular mechanisms underlying clonal diversification of the ST131 lineage.

## IMPORTANCE

*E. coli*, particularly the ST131 extraintestinal pathogenic *E. coli* (ExPEC) lineage, is an important cause of community- and hospital-acquired infections, such as urinary tract infections, surgical site infections, bloodstream infections, and sepsis. The treatment of infections caused by ExPEC has become very challenging due to the emergence of resistance to the first-line as well as the last-resort antibiotics. This study analyzes *E. coli* ST131 against three other important and globally distributed ExPEC lineages (ST38, ST405, and ST648) that also produced extended-spectrum β-lactamase (ESBL). This is perhaps the first study that employs the high-throughput whole-genome sequence-based approach to compare and study the genomic features of these four ExPEC lineages in relation to their functional properties. Findings from this study highlight the differences in the genomic coordinates of ST131 with respect to the other STs considered here. Results from this comparative genomics study can help in advancing the understanding of ST131 evolution and also offer a framework towards future developments in pathogen identification and targeted therapeutics to prevent diseases caused by this pandemic *E. coli* ST131 clone.

## INTRODUCTION

Extraintestinal pathogenic *Escherichia coli* (ExPEC) strains are a versatile variant of commensal *E. coli* demonstrating a complex genome plasticity and phylogeny (1). ExPEC strains can cause disease syndromes ranging from uncomplicated urinary tract infections to life-threatening septicemia in humans (1). Control of infections caused by ExPEC has been troublesome due to the emergence of antimicrobial resistance, particularly in the extended-spectrum β-lactamase (ESBL)-producing *E. coli* strains, which are linked with the increased rates of morbidity and mortality (2). Association of these multidrug-resistant (MDR) strains with a high rate of community- as well as hospital-acquired infections can be largely attributed to the evolution of a number of different clones with enhanced metabolic repertoires, along with increased levels of virulence and antibiotic resistance (3).

To understand the evolution of such clones, several classification schemes have been suggested, of which multilocus sequence typing (MLST) is considered to be the “gold standard” (4, 5). ESBL-producing *E. coli* ST131 was identified among thousands of sequence types (STs) or clones defined by MLST based on variations in seven housekeeping genes of *E. coli* (6, 7). Compared to other ExPEC clones, ST131 has been widely studied due to its predominant association with ExPEC infections, particularly urinary tract and bloodstream infections (8–10). This globally disseminated multidrug-resistant clone belonging to phylogenetic group B2 has also been associated with the worldwide emergence of a particular ESBL genotype, CTX-M-15 (11). Relatively higher virulence and metabolic capabilities along with resistance to wider classes of antibiotics can be attributed to widespread occurrence and persistence of this particular lineage (12, 13).

Recent reports suggest that a few other ExPEC-associated sequence types, such as ST38, ST405, and ST648, were also associated with global dissemination of CTX-M-producing *E. coli* (14). *E. coli* ST38 strains carrying CTX-M, NDM-1, and OXA-48 genes were earlier reported from several countries, such as Japan, Netherlands, South Korea, and Tanzania (15–21). Another study has reported that ST38 strains isolated from Mongolian birds were carrying the ESBL genes in their chromosomes instead of the plasmids (22). While, CTX-M-positive ST405 and ST648 were reported from different parts of the world (16, 23–28), NDM-producing ST405 and ST648 were reported from United Kingdom (29, 30). Genotype ST648 reported from companion animals was also reported to be usually associated with CTX-M (31). Comparative *in vitro* analysis of virulence determinants of strains belonging to ST38, ST131, ST405, and ST648 reported the presence of certain common virulence factor genes, such as *sat*, *iutA*, *malX*, *usp*, and *ompT*, which were associated with greater adaptability, competitiveness, and colonization capabilities (5, 32, 33).

Whole-genome-based comparative analysis provides insights into the variation in the genetic architecture within or among different species (34). While many ST131 whole-genome sequences have already been reported and analyzed worldwide, no comparative studies have yet been performed with ST38, ST405, and ST648 strains. An exhaustively comparative study on the genomic landscapes of ST131 versus other epidemiologically successful sequence types would be very pertinent to understand the evolutionary mechanisms of these STs; this therefore constituted the main focus of our study. This study included whole-genome sequencing of five ExPEC strains belonging to the four STs, which were previously obtained from western India (21). In addition, whole-genome-based comparative analysis of 139 genomes representative of strains belonging to ESBL-producing ST38, ST405, ST648, and ST131 as obtained from public as well as in-house databases was performed. Findings from this study provide an insight into the genetic-level differences in ST131 with respect to the other STs, which could be the potential basis for their global predominance in ExPEC infections.

## RESULTS

### Strain information and genome characteristics of in-house strains.

The assembly of NA023, NA081, NA090, NA101, and NA112 strains comprised 176, 223, 193, 175, and 168 contigs, respectively, with an approximate genome size of 5.2 Mb. The average G+C content was ~50%, and the average coding sequence (CDS) number of all the strains was found to be around ~5,000, amounting to a coding percentage of ~87%. The characteristics of the five genomes are presented in Table S1 in the supplemental material. While two strains, NA101 and NA112, belonged to ST131, strains NA023, NA081, and NA090 belonged to ST648, ST405, and ST38, respectively.

10.1128/mBio.01596-17.3 TABLE S1  Background data and genome characteristics of the five in-house strains NA023, NA081, NA090, NA101, and NA112 belonging to four STs considered in this study. Download TABLE S1, PDF file, 0.2 MB. Copyright © 2017 Shaik et al. 2017 Shaik et al. This content is distributed under the terms of the Creative Commons Attribution 4.0 International license.

### Data set collection for the comparative analysis.

Apart from the five in-house strains, 134 strains belonging to the four STs were selected from the public databases. Thus, the data set included 99 ST131 strains (from all three clades A, B, and C), 12 ST38, 10 ST405, and 18 ST648 comprising 139 strains in total from different isolation sources such as stool, patients suffering from urinary tract infection (UTI) and bacteremia, etc. (see Table S2 in the supplemental material). These 139 strains were used for the *in silico* comparative analysis, and five in-house strains (NA023, NA081, NA090, NA101, and NA112) were characterized by various functional assays.

10.1128/mBio.01596-17.4 TABLE S2  Information about 139 strains belonging to ST38, ST405, ST648, and ST131 considered for the comparative genomic analysis in this study. Download TABLE S2, PDF file, 0.2 MB. Copyright © 2017 Shaik et al. 2017 Shaik et al. This content is distributed under the terms of the Creative Commons Attribution 4.0 International license.

### Whole-genome-based phylogenetic analysis confirms clonality within the sequence types.

A core genome-based phylogenetic tree (see Fig. S1 in the supplemental material) showed four distinct clades emulating the MLST scheme depicting the clonality as well as distinctness at the whole-genome level. Three clades, namely, A (orange), B (pink), and C (red) as earlier reported (35, 36) were also observed within the ST131 data set. Clades of ST131 were observed to be more distant from the remaining STs, and also clades representing ST38 (purple) and ST405 (blue) were relatively closer than ST648 (green) (Fig. S1).

10.1128/mBio.01596-17.1 FIG S1  Whole-genome phylogenetic tree depicting individual STs as separate clades. Clades A, B, and C of ST131 were observed to be more distant than those of the remaining ST38, ST405, and ST648. Download FIG S1, TIF file, 2.7 MB. Copyright © 2017 Shaik et al. 2017 Shaik et al. This content is distributed under the terms of the Creative Commons Attribution 4.0 International license.

### STs exhibit similar plasmid and resistance profiles.

From the BLASTn (37) analysis of 139 strains against the downloaded Plasmid Finder database (38), it was observed that 98 out of 99 strains of ST131 harbored IncF plasmids. Among them, the in-house strains NA097 (39), NA101, and NA114 (40) harbored an IncF plasmid with pMLST profile F2:A1:B−, while NA112 had IncF with F4:A−:B− (see Table S3 in the supplemental material). These two pMLST profiles were earlier reported to be most common among the ST131 strains (41). IncF was also observed to be the major class within ST38 and ST405, where 11 out of 12 strains of ST38 and all 10 strains of ST405 harbored IncF. In the case of ST648, only 7 out of 18 genomes contained IncF. None of the strains from ST38, ST405, and ST648 had ST131-associated F2:A1:B− and F1:A2:B20 pMLST profiles. In addition to IncF, plasmid types IncHI2, IncI1, IncN, IncP, IncQ, IncB/O/K/Z, IncX1, IncX3, IncX4, and IncY were observed in lower prevalence in ST131, ST38, and ST405, while a few ST648 strains also harbored IncHI2 and IncN (Table S3).

10.1128/mBio.01596-17.5 TABLE S3  Plasmid classes observed in each of the strains belonging to ST38, ST405, ST648, and ST131. A positive hit with identity of ≥95% and query coverage of ≥90% indicates the presence of that particular class in the strain. Download TABLE S3, PDF file, 0.5 MB. Copyright © 2017 Shaik et al. 2017 Shaik et al. This content is distributed under the terms of the Creative Commons Attribution 4.0 International license.

BLASTp (37) analysis of putative protein sequences of the 139 strains against the CARD (42) database (see Fig. S2A in the supplemental material) showed that the resistance profiles of strains belonging to all four STs were very similar. The majority of the strains from ST131 (44/99) and ST405 (8/10) carried the CTX-M-15 gene, while only 2 out of 12 strains of ST38 and 6 out of 18 strains of ST648 carried this gene. Similarly, variants of TEM, SHV, and OXA were also present in most of the strains from all four STs. Other resistance genes from different classes were also present in the majority of strains, but no ST-specific pattern was apparent. The five in-house strains NA023, NA081, NA090, NA101, and NA112 were tested *in vitro* for 18 different antimicrobials belonging to six different classes, namely, β-lactam antibiotics (penicillin and cephalosporin), fluoroquinolone, aminoglycoside, tetracycline, trimethoprim/sulfonamide, and macrolide. The four strains NA101, NA023, NA081, and NA090, belonging to different sequence types ST131, ST648, ST405, and ST38, were resistant to 10, 12, 12, and 8 antibiotics, respectively. The other strain of ST131, NA112, was resistant to only six antibiotics. In-house strains NA023, NA081, NA090, and NA101 harbored the CTX-M-15 gene. The complete resistome of in-house strains is represented in Fig. S2B.

10.1128/mBio.01596-17.2 FIG S2  (A) Heat map depicting the presence of CARD resistance genes in our data set containing 139 strains belonging to ST38, ST405, ST648, and ST131 (clades A, B, and C). Black indicates a positive hit with identity of ≥70% and query coverage of ≥75%. (B) Representation of *in vitro* analysis of five in-house strains tested for 18 different antimicrobials belonging to six different classes. Download FIG S2, TIF file, 1.4 MB. Copyright © 2017 Shaik et al. 2017 Shaik et al. This content is distributed under the terms of the Creative Commons Attribution 4.0 International license.

### ST131 exhibits a relatively robust virulence profile.

Previous *in vitro* virulence studies (5) have reported that certain virulence factors, such as *sat* (secreted autotransporter toxin) and *iutA* (aerobactin siderophore receptor), were significantly present in the four genotypes ST131, ST38, ST405, and ST648, whereas the pathogenicity island marker gene *malX* was present in all but ST38. Also, certain factors were reported to be specifically present in a single genotype, such as *hra* (heat-resistant agglutinin) and group 2 capsule variant K2 in ST38, *kpsM-III* group 3 capsule in ST405, and outer membrane protease T (*ompT*) in ST131 and ST648. *sat*, the uropathogenic-specific protein gene (*usp*), and the adhesion siderophore gene (*iha*) were significantly common in ST131. A similar pattern was also observed in our data set. Virulence factor *kpsM-III* group capsule was present in 4 out of 10 strains of ST405 and in a single strain of ST648 but was missing from all 99 strains of ST131. Also, *hra*, which was reported to be commonly present in ST38, was observed in 11 out of 12 strains and few strains from other STs. *ompT*, which was earlier reported in the strains of ST131 and ST648, was observed to be present in 17/18 strains of ST648 and all 99 strains of ST131 but was missing in the remaining STs. While *usp* was exclusively present in all 99 strains of ST131, *cnf1* and *iroN* were present only in 14 and 8 ST131 strains, respectively. These three virulence factors (*usp*, *cnf1*, and *iroN*) were completely missing from ST38, ST405, and ST648. The pathogenicity island marker (*malX*) was present in all 139 strains irrespective of the STs.

**FIG 1:**
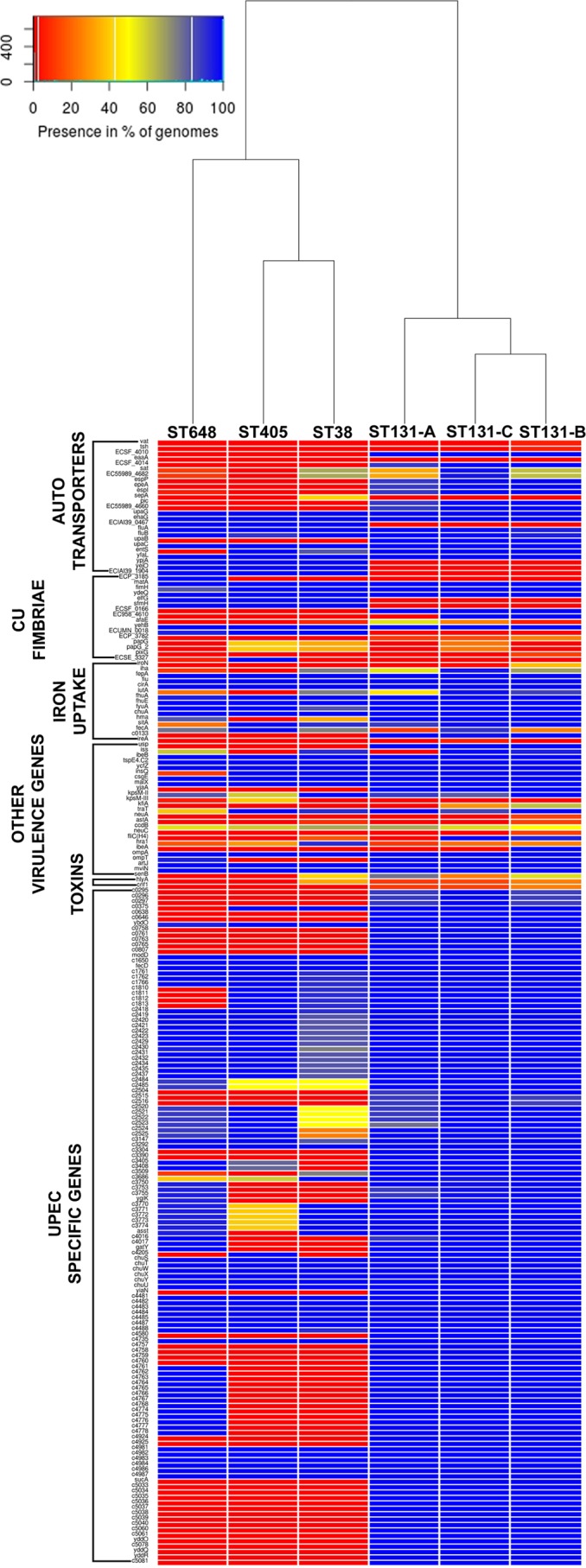
Gradient heat map depicting the frequency of virulence genes in ST38, ST405, ST648, and ST131 (clades A, B, and C). Frequency of each gene is calculated by the formula (presence in no. of strains of ST/total no. of strains in ST) × 100. Colors ranging toward red and blue depict lower and greater frequencies, respectively. Strains belonging to all three clades of ST131 harbored the majority of UPEC-specific genes, while the rest of the STs (ST38, ST405, and ST648) had them in low frequencies.

Other than this, certain other observations were captured in the heat map (Fig. 1) that illustrates the frequency of the Virulence Factor Database (VFDB) genes (35) in all four STs. Adhesion- and invasion-related genes, such as *upaG*, *ehaG*, *yfaL*, *ibeB*, and *ompA*, were present in all 139 strains, but several autotransporter genes, serine protease genes (*espP*, *epeA*, *pic*, and EC55989_4660), and many uropathogenic *E. coli* (UPEC)-specific virulence genes were absent among ST38, ST405, and ST648 strains, while they were present in the majority of ST131 strains. Few autotransporter genes, such as *ypjA*, *yejO*, and ECIAI39_1904, were missing from ST131 but were present in the remaining STs (Fig. 1).

**FIG 2:**
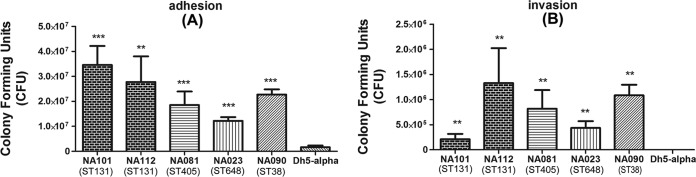
*In vitro* assays performed on five in-house strains NA023 (ST648), NA081 (ST405), NA090 (ST38), NA101 (ST131), and NA112 (ST131) belonging to all four STs considered in this study. (A) Adhesion assay. (B) Invasion assay. All strains could significantly adhere to and invade T24 bladder cell lines compared to DH5α.

The *in vitro* adhesion and invasion assays revealed that all five strains were able to adhere and invade the bladder epithelial cell line T24 compared to the negative control, *E. coli* DH5α. Strains of all four sequence types were significantly more adherent (*P* < 0.001). There were certain strain-specific differences in adherence capabilities (Fig. 2A). Similarly, the strains were also significantly more invasive than the negative control (Fig. 2B). Thus, the strains demonstrated similar pathogenic capabilities in terms of adherence and invasion to epithelial cells, irrespective of sequence types.

### Mobile genetic elements indeed define ST131.

**FIG 3:**
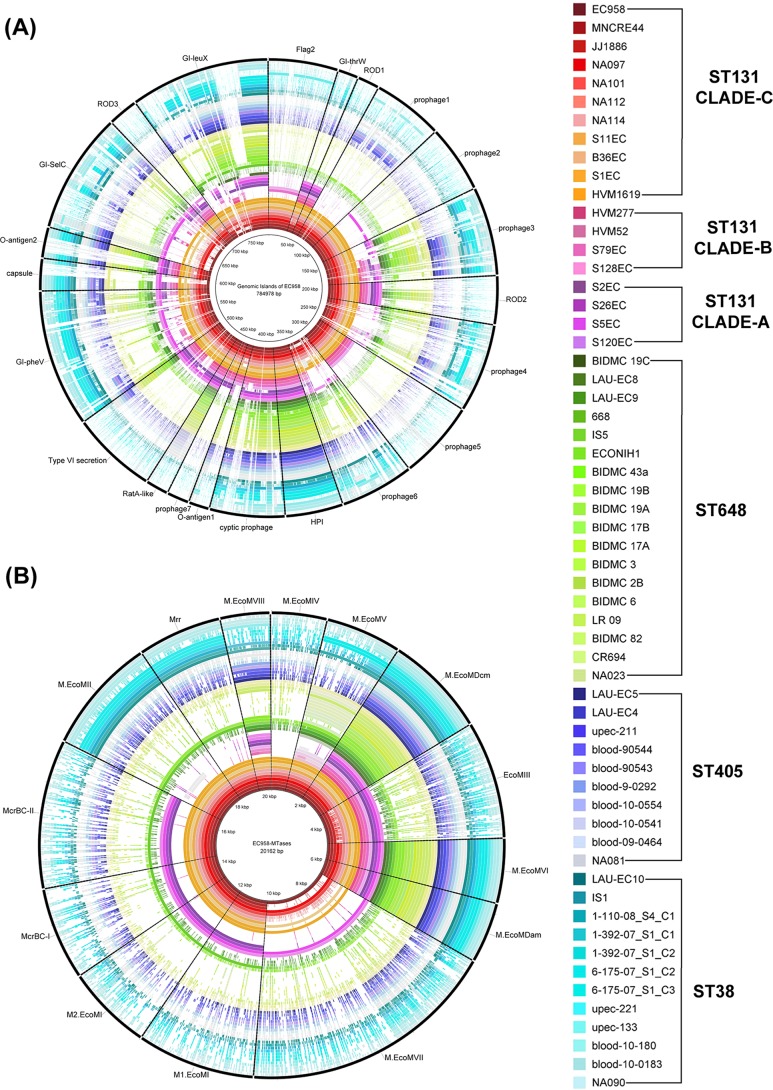
Mobile genetic elements and MTases in non-ST131 (ST38, ST405, and ST648) strains. (A) BLAST Ring Image Generator (BRIG) image showing the presence of mobile genetic elements reported in EC958 in our data set. (B) BRIG image with EC958 MTases as the reference. The majority of the mobile genetic elements and their associated MTases were found to be either missing or partially present in the non-ST131 genomes, while their presence was predominant in ST131 genomes.

BLAST Ring Image Generator (BRIG [43]) analysis (Fig. 3A) of mobile genetic elements (MGEs) revealed that the genomic islands (GIs) *GI-leuX*, *GI-pheV*, and *GI-thrW* were conserved in the majority of the ST131 strains but were either missing partially or completely from non-ST131 strains. *GI-thrW* was observed to be missing in the two in-house ST131 strains NA101 and NA112. *GI-pheV* which did not follow any conservation pattern in case of ST131 strains was also completely or partially absent in all other STs. So was the case with the other mobile genetic elements. The 31,448-bp high-pathogenicity island (HPI) from EC958, which is 99% identical to that of *Yersinia pestis* (44), was found to be the only mobile genetic element in all strains of ST131, ST405, and ST648 and 10 out of 12 strains of ST38.

A similar BRIG image (Fig. 3B) analyzing methyltransferases (MTases) revealed that chromosome-associated MTases such as M.EcoMDcm (EC958_2226), M.EcoMVI (EC958_3663), and M.EcoMDam (EC958_3778) were present in all strains of the data set. M.EcoMIII (EC958_0425), a type I restriction modification (R-M) system that is part of *GI-thrW*, was missing from all strains of ST38, ST405, and ST648 and also two ST131 strains (NA101 and NA112). Lack of *GI-thrW* in these strains as discussed above justifies the absence of this particular MTase. While M.EcoMIV (EC958_1101) encoded by prophage 2 was conserved in most of the strains of ST131 clade C strains, it was completely missing in the remaining strains. M.EcoMVII (EC958_4083) was observed to be present in only few strains of ST131 emulating the pattern of its associated mobile genetic element *GI-selC*. Other MTases, such as M1.EcoMI/M2.EcoMI (EC958_0008/EC958_0009), M.EcoMII (EC958_0078), M.EcoMV (EC958_1545), M.EcoMVIII (pEC958_A0009), McrBC (EC958_0011 and EC958_0012), and Mrr (EC958_0079) were present in almost all strains of clade C and a few other strains.

### Comparison of essential gene sets of serum resistome.

**FIG 4:**
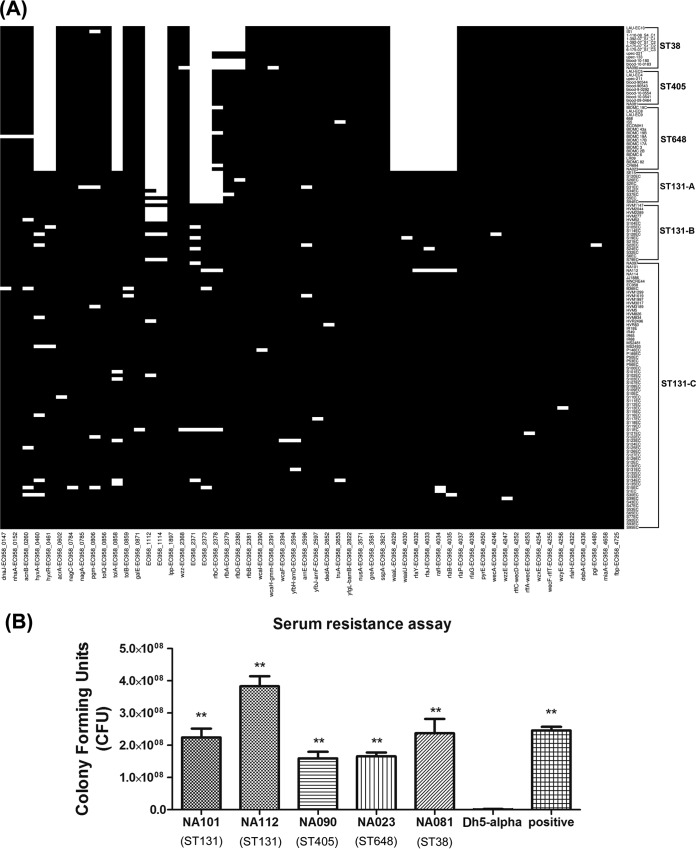
Serum resistance analysis. (A) Heat map depicting the 56 essential serum resistome genes in our data set containing 139 strains belonging to ST38, ST405, ST648, and ST131 (clades A, B, and C). Black indicates a positive hit with identity of ≥70% and query coverage of ≥75%. (B) Serum resistance assay. Genome-wide screening of serum resistance genes revealed that few essential genes were missing from non-ST131 (ST38, ST405, and ST648) genomes, but the levels of phenotypic serum resistance were found to be comparable among all four STs.

All 139 strains were analyzed for the presence of 56 essential genes belonging to the serum resistome of EC958, which have been recently defined by transposon-directed insertion site sequencing (TraDIS [45]). It was observed from the BLASTp analysis (Fig. 4A) that a few genes were missing (hit identity of <70% and query coverage of <75%) from the complete data set of ST38, ST405, and ST648, which includes EC958_0460 (*hyxA*), EC958_0461 (*hyxR*), EC958_1112, EC958_1114, EC958_2371, EC958_2373, EC958_4029 (*waaL*), EC958_4030 (*waaU* [*waaK*]), EC958_4035 (*waaB* [*rfaB*]), EC958_4032 (*waaY* [*rfaY*]), EC958_4033 (*waaJ* [*rfaJ*]), and EC958_4034 (*waaI* [*rfaI*]) (Fig. 4B), while they were present in all ST131 strains. EC958_0858 (*tolA*), belonging to the Tol-Pal system of *E. coli*, was observed to be only partially present in these strains (129 bp out of 436 bp with identity of >90%).

*In vitro* serum resistance analysis of the in-house strains NA101, NA112, NA090, NA023, and NA081 belonging to all four sequence types revealed that all strains were found to be resistant to the bactericidal activity of human serum and showed significant growth in 50% human serum in comparison to the negative control (DH5α) (Fig. 4B), irrespective of their sequence types. NA114 and NA097 were also earlier reported to be resistant to human serum (39, 40).

### Genes missing from ST131.

Core genome analysis was performed to identify the core gene content separately for each sequence type: ST38, ST131, ST405, and ST648. About 5,169, 8,189, 5,495, and 5,851 orthologous groups were identified in the cases of ST38, ST131, ST405, and ST648, respectively. Of these 4,101, 4,140, 4,362, and 4,279 belonged to their respective cores. The core protein sequences, including paralogs, were extracted from one representative strain of each ST, and another round of core genome analysis was performed using only the core protein sequences of individual STs. This analysis resulted in 4,328 orthologous groups, of which 3,472 belonged to the common core and 856 groups belonged to the accessory content.

Accessory content of the four cores, excluding the paralog clusters was further analyzed to identify any proteins that were specifically present or absent in ST131. We could not identify any ST131-specific proteins; however, 224 proteins that were specifically absent in the core of ST131 were identified. Another round of BLASTp analysis of these 224 protein sequences against the complete data set of 139 strains revealed that about 142 proteins (see Table S4 in the supplemental material) were indeed absent in all 99 strains of ST131 but were present in the remaining 40 strains belonging to ST38, ST405, and ST648.

10.1128/mBio.01596-17.6 TABLE S4  Information about the list of proteins that are specifically missing in all 99 strains of ST131 while present in the remaining 40 strains belonging to ST38, ST405, and ST648. Presence represents a positive BLASTp hit with identity of ≥70%. Download TABLE S4, PDF file, 0.3 MB. Copyright © 2017 Shaik et al. 2017 Shaik et al. This content is distributed under the terms of the Creative Commons Attribution 4.0 International license.

The functional annotation of these 142 sequences revealed that 99 proteins had a Clusters of Orthologous Groups (COG) (46) domain. These 99 proteins included membrane proteins, transcriptional regulators, transporters, toxins and antitoxins, chaperones, fimbrial proteins, and those belonging to a wide range of COG categories (Table S4).

## DISCUSSION

The enormous diversity of *E. coli* has made it the subject of innumerable comparative and phylogenetic studies in order to determine the mechanisms by which each of the subgroups/lineages has diversified and specialized. The recent emergence of the multidrug-resistant *E. coli* lineages (ST131, ST38, ST405, and ST648) as the globally dominant strains isolated from extraintestinal infections provides a new and important opportunity to understand how *E. coli* evolves and diversifies (14). Although the genomic architecture of ST131 has been widely studied, this is the first report on a genome-based comparison of ST131 strains together with three other emerging ESBL-producing ExPEC lineages: ST38, ST405, and ST648. Since *E. coli* is a very versatile organism, longitudinal studies on its genomics are very essential to understand the differences in the genomic coordinates and to understand its evolution. In this study, a data set of 5 in-house-sequenced strains along with 134 strains from the public databases belonging to these four STs was analyzed.

Clonality within the STs as depicted from the phylogenetic analysis (Fig. S1) suggests that these STs differ at the genetic level, although they are all responsible for ExPEC infections. Plasmid analysis has revealed that IncF plasmid is not just successful in ST131 as earlier reported but also most often found in the strains of the remaining STs analyzed in this study (35, 47, 48) (Table S3). The pMLST profiles F2:A1:B− and F1:A2:B20, which were commonly associated with IncF of ST131 strains (35), were not seen in any of the non-ST131 strains across the data set. Together with IncF, an intermixture of other plasmid classes, such as IncHI2, IncI1, IncN, IncP, IncQ, IncB/O/K/Z, IncX1, IncX3, IncX4, and IncY, was found among all the STs to a lesser extent. Similar to plasmid profiles, the antimicrobial resistance profiles as inferred from both genomic analysis of 139 strains and antimicrobial susceptibility assays on five in-house strains revealed that overall antimicrobial resistance was common and differed insignificantly between the four STs (Fig. S2). The presence of ESBL genes *bla*_CTX-M_, *bla*_TEM_, and *bla*_SHV_, together with several other resistance genes belonging to different classes, such as efflux pumps, pump regulators, and target inactivators, reflects the MDR genotypes of these STs (Fig. S2).

The presence of certain virulence-associated genes, such as *hra* (heat-resistant agglutinin), the *kpsM-III* group 3 capsule gene, *ompT* (outer membrane protease T), and *malx* (pathogenicity island marker) in these four STs was observed to emulate a previous study on *in vitro* virulence profiles of these four STs (5). Also, the presence of several serine proteases, autotransporters, and UPEC-specific virulence genes exclusively in the strains of ST131 was observed, which could be a reason for the predominance of ST131 strains in extraintestinal infections over any other single sequence type. The presence of genes related to adherence and invasion, such as *ibeA*, *ibeB*, *fimH*, *papG*, and *afaE*, among all the strains indicated comparable adhesion and invasion capabilities for the four STs. This supposition was validated by results of the adhesion and invasion assays on the in-house strains of all four STs (Fig. 2). Also, Wang et al. demonstrated that the components of the type 3 secretion system (T3SS) play roles in virulence and intracellular survival of an avian-pathogenic *E. coli* (APEC) strain in macrophages (49); thus, roles of such factors might also influence adhesion and invasion of ExPEC strains. In Gram-negative bacteria, the majority of serine proteases secreted by the autotransporter pathway are implicated in virulence (50). This could be the strategy of ST131 strains to enhance their virulence by consistently retaining the key serine protease autotransporters together with a unique set of virulence determinants.

Mobile genetic elements (MGEs) predominantly encode virulence and related factors (44, 51) that have been shown to play an important role in transforming UPEC strains from an acute infectious state to a chronic infectious one (52–54). An earlier study on comparative genomic analysis of ST131 strains has shown majority of the MGEs to be present in ST131 clade C (35). From our study, BRIG analysis of MGEs (previously defined in EC958 [44]) revealed that most of these regions were missing either completely or partially in ST38, ST405, and ST648 (Fig. 3A). Since EC958 belongs to ST131, it further indicates that ST131 strains have unique set of mobile genetic elements. Earlier *in silico* studies with ST131 strains (35) revealed that the presence of these regions was one of the major causes behind the diversity within the ST131 lineage. Also, MTases such as EcoMIII (EC958_0425), M.EcoMIV (EC958_1101), M.EcoMVII (EC958_4083), and M1.EcoMI/M2.EcoMI (EC958_0008/EC958_0009), which are associated with *GI-thrW*, *Phi-2*, *GI-selC*, and *GI-leuX*, respectively, were also found to be missing in strains lacking these MGEs (Fig. 3B). Three of the MTases mentioned above are part of restriction modification (R-M) systems, which are known to act as barriers for DNA exchange among species as well as within species. It would be possible that these MTases have a prominent role in segregating and shaping up the gene pool of ST131 (51). Lack of these MTases in remaining STs may indicate that they must be still open for gene exchange with other STs through HGT.

Serum resistance is one of the major survival mechanisms adopted by pathogenic *E. coli* to survive in the bloodstream of the host. BLASTp analysis of the 139 strains against 56 essential serum resistance genes of EC958 has revealed that 12 of these genes were missing from all 40 strains belonging to the non-ST131 strains but present in ST131 strains (Fig. 4A). Among these missing essential serum resistance genes, EC958_0460 (*hyxA*) and EC958_0461 (*hyxR*) were reported to have a role in regulating O-antigen chain length (45). Previous studies have also shown that EC958_0461 (*hyxR*) is linked to the suppression of nitrosactive stress response as well as important for intracellular survival within macrophages (55). EC958_1112 is one of the four glucose transferases found in the O-antigen, and the operons EC958_4029 (*waaL*), EC958_4030 (*waaU* [*waaK*]), and EC958_4035 (*waaB* [*rfaB*]) were linked to lipopolysaccharide (LPS) core biosynthesis. Mutants of all these genes except for EC958_1114 were reported to be relatively more sensitive to the serum than the wild-type genes (45). Although 12 essential serum resistance genes were missing from the non-ST131 strains, all of the five in-house strains representing the four STs exhibited similar resistance capabilities against the bactericidal activity of human serum (Fig. 4B). This may be because of the presence of uncharacterized genes in these STs, which may exhibit similar functional roles. The extra set (12 genes) of serum resistance genes identified exclusively in the ST131 lineage compared to the other three STs might help in circumventing the confounders in a natural infection state.

OrthoMCL (56) analysis of core proteins belonging to each of these four STs revealed that around 142 proteins were completely missing from all 99 strains of ST131 (Table S4) but were present in all 40 strains from ST38, ST405, and ST648. COG functional annotation of these proteins revealed that they represent a wide variety of COG functional classes, which include membrane proteins, transcriptional regulators, transcriptional chaperones, lyases, hydrogenases, etc. Also, two toxin-antitoxin modules, *mazE-mazF* and *yafO-yafN*, were found to be among the 142 missing genes of ST131. While *mazE-mazF* is known to cause programmed cell death under stress conditions, such as nutrient starvation and addition of antibiotics, *yafO-yafN* is involved in SOS response triggered by DNA damage (57). The gene coding for another important protein, diguanylate cyclase (*yddV*), involved in c-di-GMP signaling, was also among these 142 genes that were absent in the ST131 strains. It was reported earlier that this protein was found to be either truncated or completely deleted in many ExPEC strains. It was hypothesized that losing this protein would negatively regulate c-di-GMP, which may in turn enhance motility and also decrease expression of curli fibers to avoid local immune defenses (58). These observations indicate that strains of ST131 lineage utilize gene loss as a mechanism in order to fine-tune their genome for maximal fitness. The possibility of such a phenomenon has been described for some bacterial species (59).

In summary, the comparative genomic analysis of ST131 strains and other prominent non-ST131 strains (ST38, ST408, and ST648) spanning the extent of extraintestinal pathogenic *E. coli* strains provides novel insights on the evolutionary mechanisms of the ST131 lineage by revealing the molecular signatures that characterize their evolution. Our results suggest that adaptive strategies of ST131 *E. coli* strains were driven mainly by gene loss, genetic exchange, and coevolution with an antimicrobial resistance repertoire. We provide an inventory of both ST131-specific genes and those that are completely lacking in the *E. coli* ST131 lineage compared to *E. coli* genomes of other three STs (ST38, ST408, and ST648). These data will help in advancing the understanding of ST131 evolution and also offer a framework to advance future developments in pathogen identification and targeted therapeutics to prevent diseases caused by this pandemic *E. coli* ST131 clone.

## MATERIALS AND METHODS

### Strain selection and whole-genome sequencing.

The five strains NA023, NA081, NA090, NA101, and NA112, belonging to ST38, ST405, ST648, and ST131 from the collection described in reference 21, were sequenced using an Illumina Miseq sequencer with an insert size of 400 to 500 bp.

### Assembly and annotation of in-house strains.

High-quality reads obtained from NGS QC Toolkit (60) were used for *de novo* assembly of contigs using Velvet (61). The thus-generated contigs were then ordered and scaffolded using C-L-Authenticator (62), and the final draft genomes were obtained by merging these scaffolds using a series of N’s. These draft genomes were then submitted to the RAST server (63) for annotation, and the genome statistics from the resulting file were extracted using ARTEMIS (64). The sequence type (ST) of each of these strains was determined by submitting the *de novo* contigs to https://cge.cbs.dtu.dk/services/MLST/.

### Data set collection for comparative genomics. (i) Collection of 99 ST131 strains.

Apart from the two strains from the present study, 97 whole-genome sequences, including NA097, NA114, JJ1886, and EC958 from the NCBI and other ST131 genomes described by Petty et al. (35), were considered for the study.

### (ii) Collection of ST38, ST405, and ST648 strains.

As there are not many studies performed at the genomic level of these STs, an in-house pipeline was devised to extract the genomic data from NCBI. As of 8 June 2015, genomic data (both complete and incomplete) belonging to 3,051 *E. coli* strains were downloaded, and in the case of strains with incomplete data, contigs that were >500 in number were eliminated. In each genome, in-house scripts were used to extract the sequences of the seven housekeeping genes (*fum*, *adk*, *gyr*, *icd*, *pur*, *mdh*, and *rec*) and an ST number was assigned based on the combination of allelic numbers of these genes (supported by the database data downloaded from http://mlst.warwick.ac.uk/mlst/dbs/Ecoli/Downloads_HTML). Among all of the STs, 12 strains from ST38, 10 strains from ST405, and 18 strains from ST648 were separated out. The ST of each of these strains was further verified by submitting the contigs to https://cge.cbs.dtu.dk/services/MLST/. Information about all the strains used in this study is provided in Table S3.

### Phylogenomic analysis.

A core-genome-based phylogenetic tree of all 139 strains belonging to the four STs was constructed using Harvest (65), and the resulting tree was visualized using FigTree (http://tree.bio.ed.ac.uk/software/figtree/).

### Detection of plasmid classes and resistance profiles.

In case of each of these strains, BLASTn of the contigs/whole genome was performed against the data downloaded from Plasmid Finder (38). A positive hit (90% query coverage and 95% identity) indicated the presence of a particular plasmid class in respective strains.

For all of the genomes, protein sequences were predicted using GeneMarkS (66). Resistance profiles of each of these strains were obtained by performing a BLASTp analysis of the predicted protein sequences against the Comprehensive Antibiotic Resistance Database (CARD). Only the genes that had hits with 70% identity and 75% query coverage were given the status of being present. A heat map was generated using R to depict the positive hits (represented in black) in each of these genomes.

### Determination of virulence gene profile.

Virulence profiles of each of these strains were obtained by performing a BLASTn analysis of 139 genomes against the database of selected VFDB (35, 67) virulence genes. Only the genes that had hits with 70% identity and query coverage of either ≥50% or ≥800 bp were given the status of being present. The frequency of each of these virulence genes at the ST level was calculated using the formula (presence in no. of strains of ST/total no. of strains in ST) × 100. The frequencies of all of the virulence genes in each of these STs are represented in the form of a heat map using R.

### Genomic islands, MTases, and serum resistome analysis.

BRIG analysis (Fig. 3A) was performed with the data set containing 12, 10, and 18 strains belonging to ST38, ST405, and ST648 using the MGEs and MTases reported in the ST131 representative strain EC958 (44, 51) as a reference. Only 20 strains from ST131 data set belonging to all three clades A, B, and C were selected for this analysis.

A BLASTp analysis was performed to detect the presence of 56 essential serum resistance genes that were earlier reported in EC958 (45). Positive hits were defined and represented in the form of a heat map, as discussed earlier in the section “Detection of plasmid classes and resistance profiles.”

### Comparative core-accessory genome analysis.

Due to the differences in the sample sizes of each sequence type, core genes of individual sequence types were determined first. Core orthologous clusters of individual STs were identified using OrthoMCL (56, 68). The parameters for deciding orthologs such as identity and E value cutoff were set to 70% and 0.00001, respectively. The genes with less than 50 amino acids were excluded from the analysis. The clusters that contained orthologs in at least 90% of the sample size constituted the core. Genes belonging to the core orthologous groups of ST38, ST131, ST405, and ST648 were extracted from one respective representative strain.

Core genes (including recent paralogs) from the orthologous clusters of the four STs were then considered for further inter-ST core-accessory genome analysis. To identify genes that were specifically present or missing in ST131, orthologous clusters of all four ST core genes were once again determined using OrthoMCL. Orthologous clusters that had missing genes only from the ST131 core were further analyzed at the complete data set level using BLASTp as described above. A final list of genes that were present in all 40 strains belonging to ST38, ST405, and ST648 but missing from all 99 strains of ST131 was identified. COG classification and their functional annotations of all the genes of interest were determined using batch CD-search (69) against the COG database.

### *In vitro* phenotypic characterization of the strains.

All five representative strains of the four different sequence types were characterized for the phenotypic properties, such as resistance to 18 different antibiotics, ESBL production, adhesion and invasion capabilities toward human bladder epithelial (T24) cell lines, and ability to resist bactericidal activity of human serum. Resistance toward antimicrobials was determined by standard Kirby Bauer disk diffusion technique using ICOSA UTI strips (Himedia, India) as per the guidelines of CLSI, and ESBL production was tested by a double-disk synergy test (12). The adhesion and invasion assays were performed as per the standard protocols described previously (70). Briefly, T24 cells were grown to monolayer in 24-well plates and infected at a multiplicity of infection (MOI) of 10 with different strains in triplicates. After 3 h of incubation at 37°C in a CO_2_ incubator, the cells were washed three times with 1× phosphate-buffered saline (PBS) and then lysed using 0.1% Triton X-100. The lysates were collected and plated after serial dilutions. For invasion, after 3 h of incubation cells were additionally incubated for 1.5 h with medium containing 100 µg/ml of gentamicin. After incubation, cells were lysed as in adhesion step and plated and CFU counts determined for all strains. The serum resistance assay was performed in sterile 50% human serum (Pan Biotech, Germany) for 3 h as per the protocols described elsewhere (70). The growth in serum was observed in terms of CFU. The adhesion and invasion assays were performed three times, while the serum resistance assay was performed twice in triplicates. For serum resistance and the adhesion assay, the Mann-Whitney test was employed to get *P* values against the negative control (DH5α), while for the invasion assay, *P* values were obtained by employing Wilcoxon’s signed-rank test against the negative control.

### Accession number(s).

The GenBank accession numbers of the five genomes sequenced for this study are JSXK00000000 (NA023), JSXM00000000 (NA081), MVIO00000000 (NA090), JSXN00000000 (NA101), and JSXO00000000 (NA112).
