# Supplementary material for: Comparative Genomic Analysis of Globally Dominant ST131 Clone with Other Epidemiologically Successful Extraintestinal Pathogenic Escherichia coli (ExPEC) Lineages
Source: mBio. 2017 Oct 24;8(5):e01596-17. doi: 10.1128/mBio.01596-17 (PMC5654935; doi:10.1128/mBio.01596-17)
Supplement: TABLE S1 [file mbo005173554st1.pdf]

**S.Table1: Information and genomic characteristics of in-house sequenced strains**

| Strain | Disease status | Collection date | ST | # Contigs | Genome size | G+C % | CDS | Coding % | # rRNAs | # tRNAs |
|---|---|---|---|---|---|---|---|---|---|---|
| NA023 | Prostitis | 12-03-2009 | ST648 | 176 | 5192057 | 50.57 | 5073 | 86.8 | 5 | 74 |
| NA081 | UTI | 10-08-2009 | ST405 | 223 | 5238190 | 50.5 | 5051 | 86.6 | 6 | 69 |
| NA090 | Septicemia | 09-02-2009 | ST38 | 193 | 5373103 | 50.41 | 5267 | 86.2 | 20 | 89 |
| NA101 | Pyelonephritis | 05-10-2009 | ST131 | 175 | 5286008 | 50.72 | 5238 | 87.2 | 12 | 80 |
| NA112 | Pyrexia of unknown origin | 03-11-2009 | ST131 | 168 | 5148591 | 50.78 | 5081 | 87.3 | 6 | 68 |
