# Supplementary material for: Comparative Genomic Analysis of Globally Dominant ST131 Clone with Other Epidemiologically Successful Extraintestinal Pathogenic Escherichia coli (ExPEC) Lineages
Source: mBio. 2017 Oct 24;8(5):e01596-17. doi: 10.1128/mBio.01596-17 (PMC5654935; doi:10.1128/mBio.01596-17)
Supplement: TABLE S2 [file mbo005173554st2.pdf]

S.Table2: Strains used for comparative analysis

S.no	Strain Name	WGS project/ SRA Accession	Isolation source	ST
1	LAU-EC10	AYNJ01	gastrointestinal	ST38
2	IS1	CBWA01	Unknown	ST38
3	1-110-08_S4_C1	JHDK01	stool	ST38
4	1-392-07_S1_C1	JNPT01	stool	ST38
5	1-392-07_S1_C2	JNPU01	stool	ST38
6	6-175-07_S1_C2	JOMS01	stool	ST38
7	6-175-07_S1_C3	JORL01	stool	ST38
8	upec-221	JSKR01	patient with urinary tract infection	ST38
9	upec-133	JSNS01	patient with urinary tract infection	ST38
10	blood-10-180	JSPS01	blood from patient with bacteremia	ST38
11	blood-10-0183	JSQQ01	blood from patient with bacteremia	ST38
12	NA090	MVIO01	Septicemia	ST38
13	LAU-EC5	AYOG01	Gastrointestinal	ST405
14	LAU-EC4	AYOP01	Gastrointestinal	ST405
15	upec-211	JSLB01	patient with urinary tract infection	ST405
16	blood-90544	JSPE01	blood from patient with bacteremia	ST405
17	blood-90543	JSPF01	blood from patient with bacteremia	ST405
18	blood-9-0292	JSPI01	blood from patient with bacteremia	ST405
19	blood-10-0554	JSQN01	blood from patient with bacteremia	ST405
20	blood-10-0541	JSQO01	blood from patient with bacteremia	ST405
21	blood-09-0464	JSRM01	blood from patient with bacteremia	ST405
22	NA081	JSXM01	patient with Urinary tract infection	ST405
23	BIDMC 19C	AXLI01	Urine	ST648
24	LAU-EC8	AYNH01	gastrointestinal	ST648
25	LAU-EC9	AYNI01	gastrointestinal	ST648
26	668	AYQW01	Unknown	ST648
27	IS5	CBWB01	Unknown	ST648

28	ECONIH1	CP009859.1	rectal swab	ST648
29	BIDMC 43a	JAPF01	Blood Culture	ST648
30	BIDMC 19B	JAPI01	Urine	ST648
31	BIDMC 19A	JAPJ01	Bronchoalveolar Lavage	ST648
32	BIDMC 17B	JAPK01	Peritoneal Fluid	ST648
33	BIDMC 17A	JAPL01	Blood Culture	ST648
34	BIDMC 3	JAPP01	Swab, Abdominal Incision	ST648
35	BIDMC 2B	JAPQ01	Blood Culture	ST648
36	BIDMC 6	JAPV01	Bile	ST648
37	LR09	JDVF01	wastewater treatment plant	ST648
38	BIDMC 82	JJMY01	urine	ST648
39	CR694	JTGI01	urine	ST648
40	NA023	JSXK01	Prostitis	ST648
41	SE15	AP009378.1	feces	ST131-A
42	S120EC	ERR161283	Blood	ST131-A
43	S26EC	ERR161245	Urine	ST131-A
44	S2EC	ERR161235	Urine	ST131-A
45	S31EC	ERR161300	Urine	ST131-A
46	S34EC	ERR161247	Urine	ST131-A
47	S37EC	ERR161302	Urine	ST131-A
48	S5EC	ERR161236	Urine	ST131-A
49	S94EC	ERR161257	Blood	ST131-A
50	HVM1147	ERR161318	Peritoneal fluid	ST131-B
51	HVM2044	ERR161323	Blood	ST131-B
52	HVM2289	ERR161325	Urine	ST131-B
53	HVM277	ERR161315	Urine	ST131-B
54	HVM52	ERR161308	Urine	ST131-B
55	S104EC	ERR161267	Urine	ST131-B
56	S105EC	ERR161268	Urine	ST131-B
57	S114EC	ERR161277	Urine	ST131-B
58	S128EC	ERR161291	Blood	ST131-B
59	S19EC	ERR161241	Urine	ST131-B
60	S21EC	ERR161242	Urine	ST131-B
61	S22EC	ERR161243	Urine	ST131-B
62	S24EC	ERR161244	Urine	ST131-B
63	S32EC	ERR161301	Urine	ST131-B
64	S6EC	ERR161299	Urine	ST131-B
65	S79EC	ERR161305	Urine	ST131-B
66	B36EC	ERR161254	Blood	ST131-C

67	HVM1299	ERR161320	Abdominal abscess	ST131-C
68	HVM1619	ERR161321	Surgical wound	ST131-C
69	HVM1997	ERR161322	Urine	ST131-C
70	HVM3017	ERR161328	Urine	ST131-C
71	HVM3189	ERR161329	Urine	ST131-C
72	HVM5	ERR161306	Urine	ST131-C
73	HVM826	ERR161316	Blood	ST131-C
74	HVM834	ERR161317	Urine	ST131-C
75	HVR2496	ERR161326	Blood	ST131-C
76	HVR83	ERR161311	Blood	ST131-C
77	IR18E	ERR458470	Unknown	ST131-C
78	IR49	ERR458471	Unknown	ST131-C
79	IR65	ERR458472	Unknown	ST131-C
80	IR68	ERR458473	Unknown	ST131-C
81	MS2481	ERR161252	Blood	ST131-C
82	MS2493	ERR161253	Blood	ST131-C
83	P146EC	ERR161313	Rectal swab	ST131-C
84	P189EC	ERR161314	Rectal swab	ST131-C
85	P50EC	ERR161307	Rectal swab	ST131-C
86	P53EC	ERR161309	Rectal swab	ST131-C
87	P56EC	ERR161310	Rectal swab	ST131-C
88	S100EC	ERR161263	Rectal swab	ST131-C
89	S101EC	ERR161264	Rectal swab	ST131-C
90	S102EC	ERR161265	Urine	ST131-C
91	S103EC	ERR161266	Urine	ST131-C
92	S107EC	ERR161270	Urine	ST131-C
93	S108EC	ERR161271	Blood	ST131-C
94	S109EC	ERR161272	Urine	ST131-C
95	S10EC	ERR161237	Urine	ST131-C
96	S110EC	ERR161273	Urine	ST131-C
97	S111EC	ERR161274	Urine	ST131-C
98	S112EC	ERR161275	Urine	ST131-C
99	S113EC	ERR161276	Rectal swab	ST131-C
100	S115EC	ERR161278	Urine	ST131-C
101	S116EC	ERR161279	Unknown	ST131-C
102	S117EC	ERR161280	Unknown	ST131-C
103	S118EC	ERR161281	Urine	ST131-C
104	S119EC	ERR161282	Blood	ST131-C
105	S11EC	ERR161238	Urine	ST131-C

106	S121EC	ERR161284	Urine	ST131-C
107	S122EC	ERR161285	Urine	ST131-C
108	S123EC	ERR161286	Urine	ST131-C
109	S124EC	ERR161287	Urine	ST131-C
110	S125EC	ERR161288	Surgical wound	ST131-C
111	S126EC	ERR161289	Urine	ST131-C
112	S127EC	ERR161290	Urine	ST131-C
113	S129EC	ERR161292	Urine	ST131-C
114	S12EC	ERR161239	Urine	ST131-C
115	S130EC	ERR161293	Urine	ST131-C
116	S131EC	ERR161294	Urine	ST131-C
117	S132EC	ERR161295	Urine	ST131-C
118	S133EC	ERR161296	Blood	ST131-C
119	S134EC	ERR161297	Urine	ST131-C
120	S135EC	ERR161298	Blood	ST131-C
121	S15EC	ERR161240	Urine	ST131-C
122	S1EC	ERR161234	Urine	ST131-C
123	S30EC	ERR161246	Urine	ST131-C
124	S39EC	ERR161248	Unknown	ST131-C
125	S43EC	ERR161249	Unknown	ST131-C
126	S47EC	ERR161250	Unknown	ST131-C
127	S53EC	ERR161251	Unknown	ST131-C
128	S65EC	ERR161303	Urine	ST131-C
129	S77EC	ERR161304	Urine	ST131-C
130	S92EC	ERR161255	Blood	ST131-C
131	S93EC	ERR161256	Blood	ST131-C
132	S95EC	ERR161258	Blood	ST131-C
133	NA097	JSXJ01	Urine sample from patient with Septicemia	ST131-C
134	NA101	JSXN01	Pyelonephritis	ST131-C
135	NA112	JSXO01	Pyrexia of unknown origin	ST131-C
136	NA114	CP002797.2	urine	ST131-C
137	JJ1886	CP006784.1	patient with fatal urosepsis	ST131-C
138	MNCRE44	CP010876.1	Sputum of sepsis patient	ST131-C
139	EC958	HG941718.1	urine	ST131-C
