# Supplementary figures and images for: Comparative Genomic Analysis of Globally Dominant ST131 Clone with Other Epidemiologically Successful Extraintestinal Pathogenic Escherichia coli (ExPEC) Lineages
Source: mBio. 2017 Oct 24;8(5):e01596-17. doi: 10.1128/mBio.01596-17 (PMC5654935; doi:10.1128/mBio.01596-17)

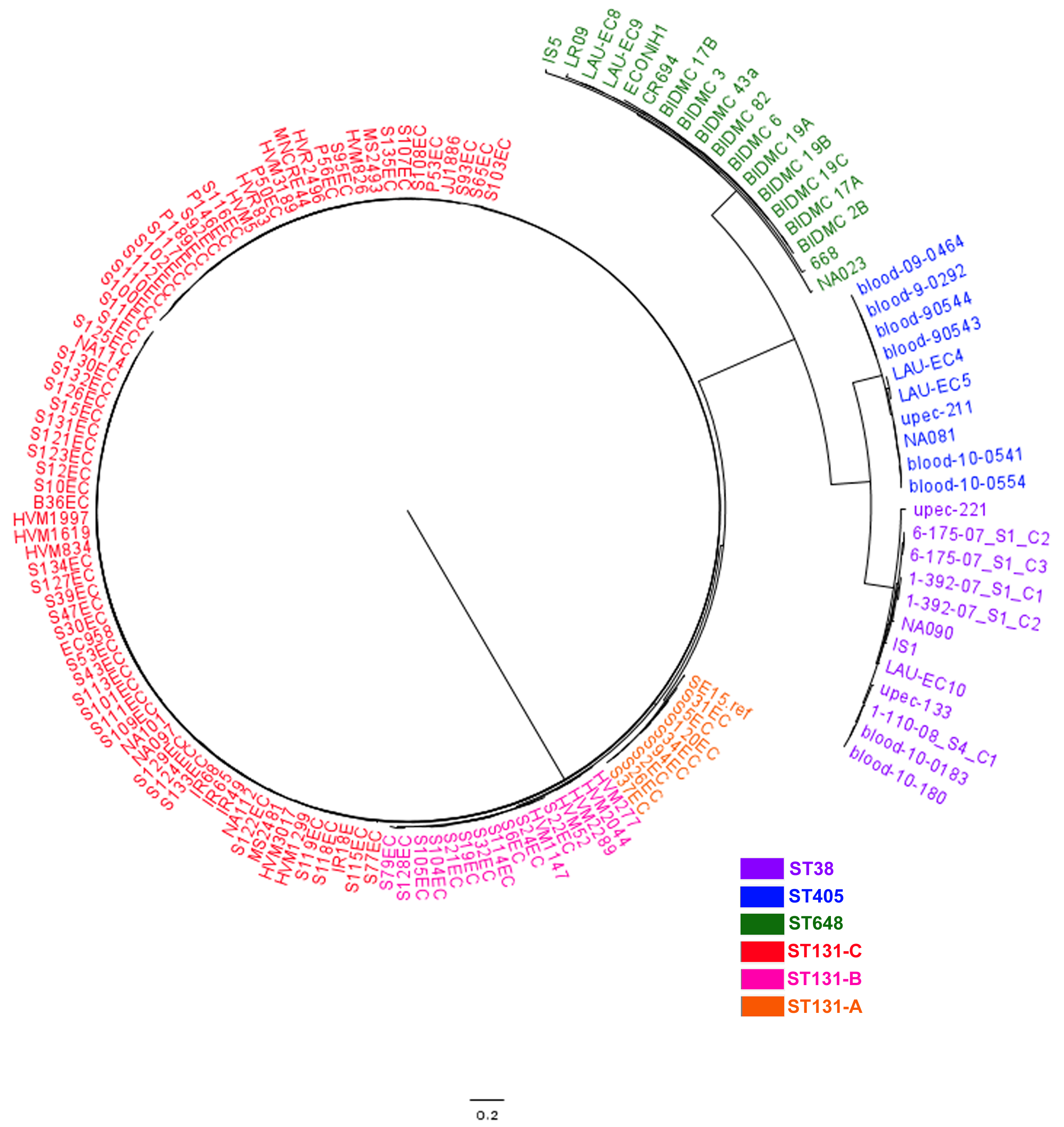

Supplement: FIG S1 [file mbo005173554sf1.tif]

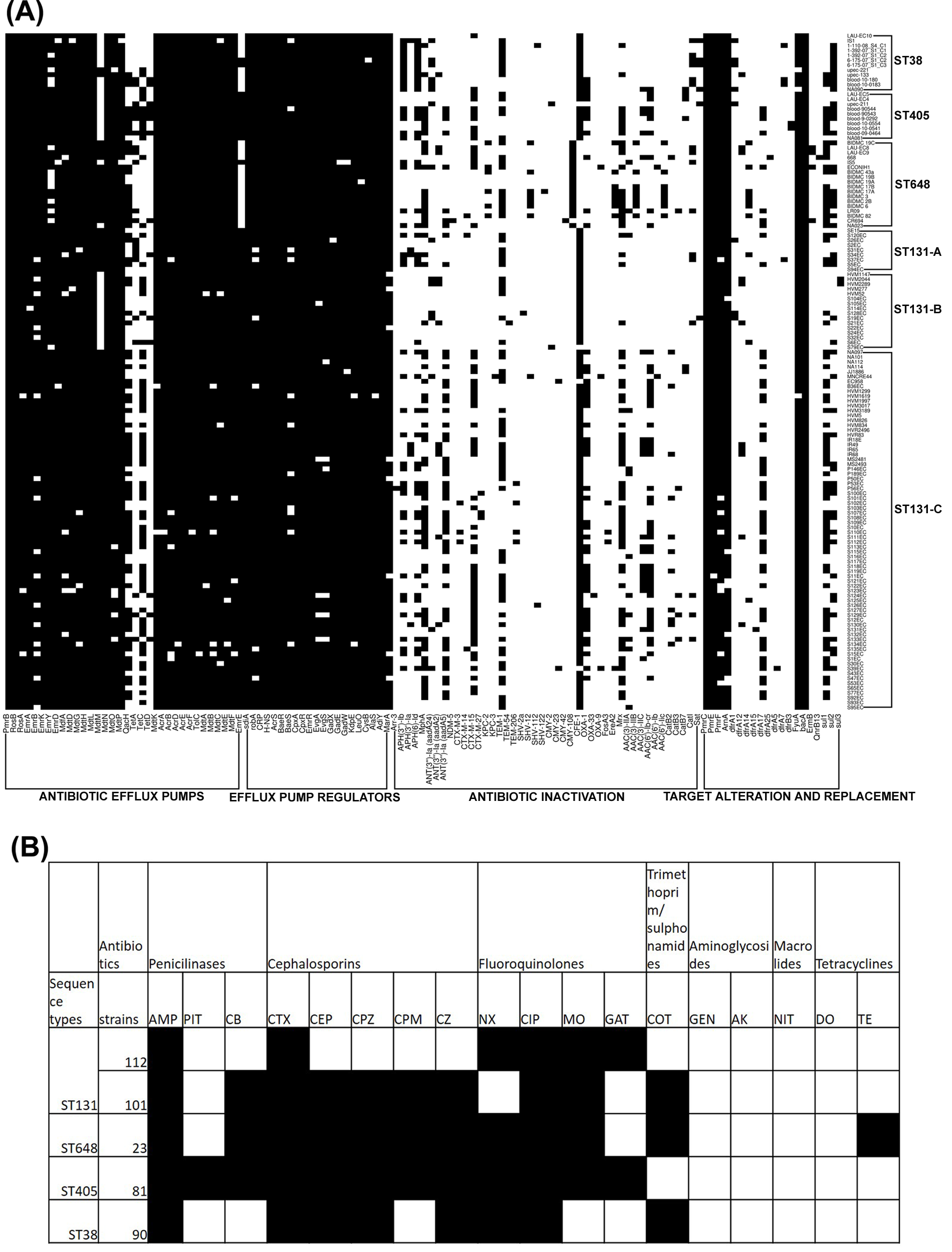

Supplement: FIG S2 [file mbo005173554sf2.tif]
