# Supplementary material for: Comparative Genomic Analysis of Globally Dominant ST131 Clone with Other Epidemiologically Successful Extraintestinal Pathogenic Escherichia coli (ExPEC) Lineages
Source: mBio. 2017 Oct 24;8(5):e01596-17. doi: 10.1128/mBio.01596-17 (PMC5654935; doi:10.1128/mBio.01596-17)
Supplement: TABLE S3 [file mbo005173554st3.pdf]

S.Table3: Plasmid profiles of 139 strains

S.No	Strain	IncF	IncHI2	IncI1	IncN	IncP	IncQ	IncB/O/K/Z	IncX1	IncX3	IncX4	IncY
	**ST38**											
1	upec-221	-	-	-	-	P	P	-	-	-	-	-
2	6-175-07_S1_C3	[F1:A-:B-]	-	-	-	-	-	-	-	-	-	-
3	IS1	[F1:A-:B23]	-	-	-	-	-	-	-	-	-	-
4	6-175-07_S1_C2	[F1:A-:B33*]	-	-	-	-	-	-	-	-	-	-
5	1-392-07_S1_C1	[F1:A-:B33]	-	-	-	-	-	-	-	-	-	-
6	1-392-07_S1_C2	[F1:A-:B33]	-	-	-	-	-	-	-	-	-	-
7	NA090	[F1:A-:B33]	-	-	-	-	-	-	-	-	-	-
8	1-110-08_S4_C1	[F2:A-:B10]	-	-	-	-	-	-	-	-	-	-
9	LAU-EC10	[F29:A4*:B10]	-	-	-	-	-	-	-	-	-	-
10	upec-133	[F31:A4:B37*]	-	-	-	-	-	-	-	-	-	-
11	blood-10-180	[F51:A-:B10]	-	-	-	-	-	-	-	-	-	-
12	blood-10-0183	[F51:A-:B10]	-	-	-	-	-	-	-	-	-	-
	**ST405**											
1	upec-211	[F1:A1:B-]	-	P	-	-	-	P	-	-	-	-
2	blood-90543	[F1*:A1:B16]	-	-	-	-	-	P	-	-	-	-
3	blood-90544	[F1:A1:B16]	-	-	-	-	-	P	-	-	-	-
4	blood-9-0292	[F1:A1:B16]	-	-	-	-	-	-	-	-	-	-
5	blood-09-0464	[F1:A1:B16]	-	-	-	-	-	-	-	-	-	-
6	blood-10-0554	[F24:A-:B6]	-	-	-	-	-	-	-	-	-	-
7	blood-10-0541	[F24:A-:B6]	-	-	-	-	-	-	-	-	-	P
8	NA081	[F24:A-:B6]	-	-	-	-	-	-	-	-	-	P
9	LAU-EC4	[F36:A1:B10]	-	-	-	-	-	-	-	-	-	-
10	LAU-EC5	[F36:A1:B10]	-	-	-	-	-	-	-	-	-	-
	**ST648**											
1	BIDMC 19C	-	P	-	P	-	-	-	-	-	P	-

2	668	-	-	-	P	-	-	P	-	-	-	-
3	BIDMC 43a	-	P	-	-	-	-	-	-	-	-	-
4	BIDMC 19B	-	-	-	-	-	-	-	-	-	-	-
5	BIDMC 19A	-	-	-	-	-	-	-	-	-	-	-
6	BIDMC 17B	-	-	-	-	-	-	-	-	-	-	-
7	BIDMC 17A	-	P	-	P	-	-	-	-	-	-	-
8	BIDMC 3	-	P	-	P	-	-	-	-	-	-	-
9	BIDMC 2B	-	P	-	P	-	-	-	-	-	P	-
10	BIDMC 6	-	P	-	P	-	-	-	-	-	P	-
11	BIDMC 82	-	P	-	P	-	-	P	-	-	P	-
12	IS5	[F1:A1:B-]	-	-	-	-	-	P	-	-	-	-
13	ECONIH1	[F1:A1:B1]	-	-	P	-	-	-	-	-	-	-
14	LR09	[F1:A1:B1]	-	-	-	-	-	-	-	-	-	P
15	NA023	[F31:A4:B1]	-	-	-	-	-	-	-	-	-	-
16	CR694	[F36:A-:B32]	-	P	-	-	-	-	-	P	P	-
17	LAU-EC8	[F48:A1:B49]	-	-	-	-	-	-	-	-	-	-
18	LAU-EC9	[F48:A1:B49]	-	-	-	-	-	-	-	-	-	-
	**ST131**											
1	HVM5	-	-	-	-	-	-	-	-	-	-	-
2	S11EC	[F-:A1:B-]	-	-	-	-	-	P	-	-	-	-
3	S1EC	[F-:A1:B-]	-	-	-	-	-	-	-	-	-	-
4	HVR83	[F-:A2:B20]	-	P	-	-	-	-	P	-	P	-
5	S103EC	[F1:A-:B23]	-	-	-	-	-	-	-	-	-	-
6	S65EC	[F1:A-:B23]	-	-	-	-	-	-	-	-	-	-
7	S34EC	[F1:A1:B1]	-	-	-	-	P	-	-	-	-	-
8	S37EC	[F1:A1:B1]	-	-	-	-	-	-	-	-	-	-
9	S26EC	[F1:A1:B23]	-	-	-	-	-	-	-	-	-	-
10	S2EC	[F1:A1:B23]	-	-	-	-	-	-	-	-	-	-
11	S31EC	[F1:A1:B23]	-	-	-	-	-	-	-	-	-	-

12	S110EC	[F1:A2:B-]	-	-	-	-	-	-	-	-	-	-
13	S116EC	[F1:A2:B-]	-	-	-	-	-	-	-	-	-	-
14	P146EC	[F1:A2:B20*]	-	-	-	-	-	P	-	-	-	-
15	MNCRE44	[F1:A2:B20]	-	-	-	-	-	-	-	P	P	-
16	HVM3189	[F1:A2:B20]	-	-	-	-	-	-	-	-	-	-
17	HVM826	[F1:A2:B20]	-	-	-	-	-	-	-	-	-	-
18	HVR2496	[F1:A2:B20]	-	-	-	-	-	-	-	-	-	-
19	MS2493	[F1:A2:B20]	-	-	-	-	-	-	-	-	-	-
20	P189EC	[F1:A2:B20]	-	-	-	-	-	-	-	-	-	-
21	P50EC	[F1:A2:B20]	-	-	-	-	-	P	-	-	-	-
22	P53EC	[F1:A2:B20]	-	-	-	-	-	-	-	-	P	-
23	P56EC	[F1:A2:B20]	-	-	-	-	-	-	-	-	-	-
24	S100EC	[F1:A2:B20]	-	-	-	-	-	-	-	-	-	-
25	S102EC	[F1:A2:B20]	-	-	-	-	-	-	-	-	-	-
26	S107EC	[F1:A2:B20]	-	-	-	-	-	-	-	-	-	-
27	S108EC	[F1:A2:B20]	-	-	-	-	-	-	-	-	-	-
28	S112EC	[F1:A2:B20]	-	-	-	-	-	-	-	-	-	-
29	S120EC	[F1:A2:B20]	-	-	-	-	-	-	-	-	-	-
30	S135EC	[F1:A2:B20]	-	-	-	-	-	-	-	-	-	-
31	S92EC	[F1:A2:B20]	-	-	-	-	-	-	-	-	P	-
32	S95EC	[F1:A2:B20]	-	-	-	-	-	-	-	-	-	-
33	S117EC	[F10:A-:B-]	-	-	-	-	-	-	P	-	-	-
34	S131EC	[F16:A-:B1]	-	-	-	P	-	-	-	-	P	-
35	HVM2044	[F2:A-:B1]	-	P	-	-	-	-	-	-	-	-
36	HVM2289	[F2:A-:B1]	-	P	-	-	-	-	-	-	-	-
37	HVM277	[F2:A-:B1]	-	-	-	-	-	-	-	-	-	-
38	HVM52	[F2:A-:B1]	-	P	-	-	-	P	-	-	-	-
39	NA097	[F2:A1:B-]	-	-	-	-	-	-	-	-	-	-
40	NA101	[F2:A1:B-]	-	-	-	-	-	-	-	-	-	-
41	NA114	[F2:A1:B-]	-	-	-	-	-	-	-	-	-	-

42	JJ1886	[F2:A1:B-]	-	-	-	-	-	-	-	-	-	-
43	EC958	[F2:A1:B-]	-	-	-	-	-	-	-	-	-	-
44	B36EC	[F2:A1:B-]	-	-	-	-	-	-	-	-	-	-
45	HVM1619	[F2:A1:B-]	-	-	-	-	-	-	-	-	P	-
46	HVM1997	[F2:A1:B-]	-	-	-	-	-	-	-	-	-	-
47	HVM834	[F2:A1:B-]	-	-	-	-	-	-	P	-	P	-
48	S101EC	[F2:A1:B-]	-	-	-	-	-	-	-	-	-	-
49	S109EC	[F2:A1:B-]	-	-	-	-	-	P	-	-	-	-
50	S10EC	[F2:A1:B-]	-	-	-	-	-	-	-	-	-	-
51	S113EC	[F2:A1:B-]	-	-	-	-	-	-	-	-	-	P
52	S121EC	[F2:A1:B-]	-	-	-	-	-	-	-	-	-	-
53	S123EC	[F2:A1:B-]	-	-	-	-	-	-	-	-	-	-
54	S126EC	[F2:A1:B-]	-	-	-	-	-	-	-	-	-	-
55	S127EC	[F2:A1:B-]	-	-	-	-	-	-	-	-	P	-
56	S12EC	[F2:A1:B-]	-	-	-	-	-	-	-	-	-	-
57	S132EC	[F2:A1:B-]	-	-	-	-	-	-	-	-	-	-
58	S134EC	[F2:A1:B-]	-	-	-	-	-	-	-	-	-	-
59	S15EC	[F2:A1:B-]	-	-	-	-	-	-	P	-	-	-
60	S30EC	[F2:A1:B-]	-	-	-	-	-	-	-	-	-	-
61	S39EC	[F2:A1:B-]	-	-	P	-	-	-	-	-	P	-
62	S47EC	[F2:A1:B-]	-	-	-	-	-	-	-	-	-	-
63	S53EC	[F2:A1:B-]	-	-	-	-	-	-	-	-	-	-
64	S77EC	[F2:A1:B-]	-	-	-	-	-	-	-	-	-	-
65	S93EC	[F2:A1:B-]	-	-	-	-	-	-	-	-	-	-
66	S125EC	[F2:A6*:B1]	-	-	-	-	-	-	-	-	P	P
67	IR49	[F22:A1:B20]	-	-	-	-	-	-	-	-	-	-
68	IR65	[F22:A1:B20]	-	-	-	-	-	-	-	-	-	-
69	S111EC	[F22:A1:B20]	-	-	-	-	-	-	-	-	-	-
70	S122EC	[F22:A1:B20]	-	-	-	-	-	-	-	-	-	-
71	S130EC	[F22:A1:B20]	-	-	-	-	-	-	-	-	-	-

72	S128EC	[F24:A-:B40]	-	P	-	P	-	-	-	-	-	-
73	SE15	[F29:A-:B10]	-	-	-	-	-	-	-	-	-	-
74	HVM1147	[F29:A-:B10]	-	-	-	-	-	-	-	-	-	-
75	S104EC	[F29:A-:B10]	-	-	-	-	-	-	-	-	-	-
76	S105EC	[F29:A-:B10]	-	-	-	-	-	-	-	-	P	-
77	S114EC	[F29:A-:B10]	-	-	-	-	-	-	-	-	-	-
78	S21EC	[F29:A-:B10]	-	-	-	-	-	-	-	-	-	-
79	S22EC	[F29:A-:B10]	-	-	-	-	-	-	-	-	-	-
80	S24EC	[F29:A-:B10]	-	-	-	-	-	-	-	-	-	-
81	S5EC	[F29:A-:B10]	-	-	-	-	-	-	-	-	-	-
82	S94EC	[F29:A-:B10]	-	-	-	-	-	-	-	-	-	-
83	HVM3017	[F31:A4:B1]	-	-	-	-	-	-	-	-	-	-
84	IR18E	[F31:A4:B1]	-	-	-	-	-	-	-	-	-	-
85	IR68	[F36:A1:B20]	-	-	-	-	-	-	-	-	-	-
86	MS2481	[F36:A1:B20]	-	-	-	-	-	-	-	-	-	-
87	HVM1299	[F36:A4:B1]	-	-	-	-	-	-	-	-	-	-
88	S115EC	[F36:A4:B1]	-	-	-	-	-	-	-	-	-	-
89	S118EC	[F36:A4:B1]	-	-	-	-	-	-	-	-	-	-
90	S119EC	[F36:A4:B1]	-	-	-	-	-	-	-	-	-	-
91	NA112	[F4:A-:B-]	-	-	-	-	-	-	-	-	P	-
92	S79EC	[F4:A-:B1]	-	P	-	-	-	-	-	-	-	-
93	S19EC	[F4:A-:B10]	-	-	-	-	-	P	P	-	P	-
94	S124EC	[F48:A1:B49]	-	-	-	-	P	-	-	-	P	P
95	S129EC	[F48:A1:B49]	-	-	-	-	P	-	-	-	P	P
96	S133EC	[F48:A1:B49]	-	-	-	-	P	-	-	-	-	-
97	S43EC	[F51*:A-:B-]	-	-	-	-	-	-	-	-	-	-
98	S32EC	[F51:A-:B10]	-	-	-	-	-	-	-	-	-	-
99	S6EC	[F51:A-:B10]	-	-	P	-	-	-	-	-	-	-

‘P’ indicates the presence of that particular plasmid class in the strain while ‘-‘indicates its absence
