# Supplementary material for: Comparative Genomic Analysis of Globally Dominant ST131 Clone with Other Epidemiologically Successful Extraintestinal Pathogenic Escherichia coli (ExPEC) Lineages
Source: mBio. 2017 Oct 24;8(5):e01596-17. doi: 10.1128/mBio.01596-17 (PMC5654935; doi:10.1128/mBio.01596-17)
Supplement: TABLE S4 [file mbo005173554st4.pdf]

S.Table4: List of orthologous proteins missing from ST131 strains

S.no	UNIPROT ID	Function	COG ID	COG class	COG Category
1	V8KDQ1	Lipopolysaccharide 1,2-glucyltransferase	COG1442	[M] Cell wall/membrane/envelope biogenesis	Cellular Processes And Signaling
2	V8JY35	D-alanyl-D-alanine dipeptidase	COG2173	[M] Cell wall/membrane/envelope biogenesis	Cellular Processes And Signaling
3	V8KE30	Betaine/carnitine/choline BCCT transporter	COG1292	[M] Cell wall/membrane/envelope biogenesis	Cellular Processes And Signaling
4	V8KDC7	Beta-1,4-galactyltransferase	COG3306	[M] Cell wall/membrane/envelope biogenesis	Cellular Processes And Signaling
5	V8K6U2	Type-1 fimbrial protein subunit A	COG3539	[N] Cell motility	Cellular Processes And Signaling
6	V8K634	Fimbrial protein	COG3539	[N] Cell motility	Cellular Processes And Signaling
7	V8KUT9	Fimbrial protein	COG3539	[N] Cell motility	Cellular Processes And Signaling
8	V8KSH5	Fimbrial protein	COG3539	[N] Cell motility	Cellular Processes And Signaling
9	V8KT34	Fimbrial protein	COG3539	[N] Cell motility	Cellular Processes And Signaling
10	V8KUU3	Fimbrial protein	COG3539	[N] Cell motility	Cellular Processes And Signaling
11	V8K5N9	Adhesin	COG3539	[N] Cell motility	Cellular Processes And Signaling
12	V8K6T9	Membrane protein	COG0425	[O] Posttranslational modification, protein turnover, chaperones	Cellular Processes And Signaling
13	V8K087	Diguanylate cyclase yddV	COG2199	[T] Signal transduction mechanisms	Cellular Processes And Signaling
14	V8KAJ7	Antitoxin MazE	COG2336	[T] Signal transduction mechanisms	Cellular Processes And Signaling
15	V8K746	Uncharacterized protein	COG3521	[U] Intracellular trafficking, secretion, and vesicular transport	Cellular Processes And Signaling
16	V8K7E7	Type VI secretion protein	COG3518	[U] Intracellular trafficking, secretion, and vesicular transport	Cellular Processes And Signaling
17	V8K4Y2	Membrane protein	COG3455	[U] Intracellular trafficking, secretion, and vesicular transport	Cellular Processes And Signaling
18	V8K751	CAD protein	COG3515	[U] Intracellular trafficking, secretion, and vesicular transport	Cellular Processes And Signaling
19	V8K349	UPF0214 protein YfeW	COG1680	[V] Defense mechanisms	Cellular Processes And Signaling

20	V8KAZ7	Toxin mazF	COG2337	[V] Defense mechanisms	Cellular Processes And Signaling
21	V8KFX2	CRISPR-associated endonuclease Cas1	COG1518	[V] Defense mechanisms	Cellular Processes And Signaling
22	V8JSL0	Antitoxin YafN	COG2161	[V] Defense mechanisms	Cellular Processes And Signaling
23	V8K7W1	Molecular chaperone	COG3121	[W] Extracellular structures	Cellular Processes And Signaling
24	V8KUP8	Fimbrial protein	COG3121	[W] Extracellular structures	Cellular Processes And Signaling
25	V8KUP6	Fimbrial chaperone BcfG	COG3121	[W] Extracellular structures	Cellular Processes And Signaling
26	V8JKS1	XRE family transcriptional regulator	COG2944	[K] Transcription	Information Storage And Processing
27	V8KUZ3	Uncharacterized protein	COG2944	[K] Transcription	Information Storage And Processing
28	V8K3J8	Uncharacterized protein	COG4886	[K] Transcription	Information Storage And Processing
29	V8K7W6	Transcriptional regulator	COG2207	[K] Transcription	Information Storage And Processing
30	V8KFV9	Transcriptional regulator	COG1846	[K] Transcription	Information Storage And Processing
31	V8KG32	Transcriptional regulator	COG1609	[K] Transcription	Information Storage And Processing
32	V8KCQ7	LysR family transcriptional regulator	COG0583	[K] Transcription	Information Storage And Processing
33	V8KVE3	LysR family transcriptional regulator	COG0583	[K] Transcription	Information Storage And Processing
34	V8KFC2	LysR family transcriptional regulator	COG0583	[K] Transcription	Information Storage And Processing
35	V8K0Z8	HTH-type transcriptional regulator MurR	COG1737	[K] Transcription	Information Storage And Processing
36	V8KIN4	GntR family transcriptional regulator	COG2188	[K] Transcription	Information Storage And Processing
37	V8K2V1	Transpase	COG5464	[L] Replication, recombination and repair	Information Storage And Processing
38	V8JLS9	Transpase	COG5464	[L] Replication, recombination and repair	Information Storage And Processing
39	V8KCB7	ATP-dependent helicase	COG1201	[L] Replication, recombination and repair	Information Storage And Processing
40	V8K144	Hydrogenase-4 subunit E	COG4237	[C] Energy production and conversion	Metabolism
41	V8K394	Hydrogenase 3 membrane subunit	COG0650	[C] Energy production and conversion	Metabolism
42	V8KTL4	Glutamate symport transmembrane protein	COG1301	[C] Energy production and conversion	Metabolism
43	V8K3H5	Formate hydrogenlyase subunit 7	COG3260	[C] Energy production and conversion	Metabolism

44	V8K1L5	Formate hydrogenlyase subunit 2	COG1142	[C] Energy production and conversion	Metabolism
45	V8KF75	Dioxygenase subunit beta	COG1018	[C] Energy production and conversion	Metabolism
46	V8KMG1	C4-dicarboxylate ABC transporter	COG3069	[C] Energy production and conversion	Metabolism
47	V8K398	(4Fe-4S)-binding protein	COG1143	[C] Energy production and conversion	Metabolism
48	V8KT59	Transporter	COG0531	[E] Amino acid transport and metabolism	Metabolism
49	V8JYJ9	Peptide ABC transporter substrate-binding protein	COG0747	[E] Amino acid transport and metabolism	Metabolism
50	V8KV94	Methylaspartate ammonia-lyase	COG3799	[E] Amino acid transport and metabolism	Metabolism
51	V8KU99	Glutamate mutase epsilon subunit	COG4865	[E] Amino acid transport and metabolism	Metabolism
52	V8KFK2	Uncharacterized protein	COG0366	[G] Carbohydrate transport and metabolism	Metabolism
53	V8KJM8	PTS alpha-glucide transporter subunit IIBC	COG1263	[G] Carbohydrate transport and metabolism	Metabolism
54	V8K583	MFS transporter	COG2814	[G] Carbohydrate transport and metabolism	Metabolism
55	V8KJR6	6-phpho-alpha-glucidase	COG1486	[G] Carbohydrate transport and metabolism	Metabolism
56	V8KIC3	Probable UbiX-like flavin prenyltransferase	COG0163	[H] Coenzyme transport and metabolism	Metabolism
57	V8KI60	Phenolic acid decarboxylase subunit C	COG0043	[H] Coenzyme transport and metabolism	Metabolism
58	V8KV99	Cob(I)alamin adenolsyltransferase/cobinamide ATP-dependent adenolsyltransferase	COG2109	[H] Coenzyme transport and metabolism	Metabolism
59	V8KT09	Glutamate mutase sigma subunit	COG2185	[I] Lipid transport and metabolism	Metabolism
60	V8KFB6	3-phenylpropionate/cinnamic acid dioxygenase ferredoxin--NAD(+) reductase component	COG0446	[I] Lipid transport and metabolism	Metabolism
61	V8K8D7	Uncharacterized protein	COG1629	[P] Inorganic ion transport and metabolism	Metabolism
62	V8K4L2	Tautomerase PptA	COG1942	[Q] Secondary metabolites biosynthesis, transport and catabolism	Metabolism

63	V8KDZ8	3-phenylpropionate/cinnamic acid dioxygenase subunit beta	COG5517	[Q] Secondary metabolites biosynthesis, transport and catabolism	Metabolism
64	V8KGH6	Uncharacterized protein	COG3468	-	Multiple-Classes
65	V8K624	Type VI secretion system protein ImpI	COG3456	-	Multiple-Classes
66	V8K1M2	Transcriptional regulator	COG3604	-	Multiple-Classes
67	V8KD21	Tartrate dehydrogenase	COG0473	-	Multiple-Classes
68	V8JZ57	Peptide ABC transporter permease	COG0601	-	Multiple-Classes
69	V8K3H0	Oxidoreductase	COG0651	-	Multiple-Classes
70	V8K3F4	NADP-dependent oxidoreductase	COG2130	-	Multiple-Classes
71	V8K4J6	Methyl-accepting chemotaxis protein	COG0840	-	Multiple-Classes
72	V8KS91	Methyl-accepting chemotaxis protein	COG0840	-	Multiple-Classes
73	V8K1L8	Hydrogenase	COG0651	-	Multiple-Classes
74	V8K263	Hydantoin racemase	COG0651	-	Multiple-Classes
75	V8KEY2	Dioxygenase	COG4638	-	Multiple-Classes
76	V8K5Y2	Adhesin	COG5295	-	Multiple-Classes
77	V8KEC3	3-phenylpropionate/cinnamic acid dioxygenase subunit alpha	COG4638	-	Multiple-Classes
78	V8KGG9	3-phenylpropionate/cinnamic acid dioxygenase ferredoxin subunit	COG2146	-	Multiple-Classes
79	V8KG59	3-phenylpropionate-dihydrodiol/cinnamic acid-dihydrodiol dehydrogenase	COG1028	-	Multiple-Classes
80	V8KHE5	Invasion protein	COG0745	[K] Transcription, [T] Signal transduction mechanisms	Multiple-Classes
81	V8K6U7	Transcriptional regulator	COG2197	[T] Signal transduction mechanisms, [K] Transcription	Multiple-Classes
82	V8KL53	Uncharacterized protein	COG4804	[R] General function prediction only	Poorly Characterized
83	V8KH81	Multidrug ABC transporter ATP-binding protein	COG4178	[R] General function prediction only	Poorly Characterized

84	V8K6I9	Membrane protein	COG2391	[R] General function prediction only	Poorly Characterized
85	V8K9C1	Lyase	COG1413	[R] General function prediction only	Poorly Characterized
86	V8KIM2	Uncharacterized protein	COG5435	[S] Function unknown	Poorly Characterized
87	V8KB66	Uncharacterized protein	COG4453	[S] Function unknown	Poorly Characterized
88	V8KHN2	Uncharacterized protein	COG3111	[S] Function unknown	Poorly Characterized
89	V8KTZ8	Toxin RelE	COG4737	[S] Function unknown	Poorly Characterized
90	V8JV47	Membrane protein	COG3152	[S] Function unknown	Poorly Characterized
91	V8KN72	Uncharacterized protein	COG1357	[S] Function unknown	Poorly Characterized
92	V8KG33	Uncharacterized protein	-	-	-
93	V8KQH5	Uncharacterized protein	-	-	-
94	V8KAN6	Uncharacterized protein	-	-	-
95	V8KCJ2	Uncharacterized protein	-	-	-
96	V8KJU4	Uncharacterized protein	-	-	-
97	V8K5J4	Uncharacterized protein	-	-	-
98	V8KAC5	Uncharacterized protein	-	-	-
99	V8KU12	Uncharacterized protein	-	-	-
100	V8KSP2	Uncharacterized protein	-	-	-
101	V8K756	Uncharacterized protein	-	-	-
102	V8KVD3	Uncharacterized protein	-	-	-
103	V8KV88	Uncharacterized protein	-	-	-
104	V8JR73	Uncharacterized protein	-	-	-
105	V8JR03	Uncharacterized protein	-	-	-
106	V8K1W4	Uncharacterized protein	-	-	-
107	V8JVI1	Uncharacterized protein	-	-	-
108	V8KIP7	Uncharacterized protein	-	-	-
109	V8KJP2	Uncharacterized protein	-	-	-
110	V8KI19	Uncharacterized protein	-	-	-
111	V8K8B8	Uncharacterized protein	-	-	-
112	V8JX34	Uncharacterized protein	-	-	-
113	V8KHM0	Uncharacterized protein	-	-	-
114	V8K1A9	Uncharacterized protein	-	-	-
115	V8K832	Uncharacterized protein	-	-	-
116	V8KAI7	Uncharacterized protein	-	-	-

117	V8K997	Uncharacterized protein	-	-	-
118	V8KDD8	Uncharacterized protein	-	-	-
119	V8K7F9	Uncharacterized protein	-	-	-
120	V8JV31	Uncharacterized protein	-	-	-
121	V8K619	Uncharacterized protein	-	-	-
122	V8K629	Type VI secretion protein	-	-	-
123	V8KIH3	Type III effector protein	-	-	-
124	V8KHG4	Type III effector protein	-	-	-
125	V8KHR0	Type III effector	-	-	-
126	V8KJG9	Type III effector	-	-	-
127	V8KJQ2	Type III cell invasion protein SipB	-	-	-
128	V8JSN6	Toxin YafO	-	-	-
129	V8JLC1	Toxin RelE	-	-	-
130	V8KID8	SsRNA endonuclease	-	-	-
131	V8K633	Membrane protein	-	-	-
132	V8K1E3	Membrane protein	-	-	-
133	V8KDP0	Membrane protein	-	-	-
134	V8K1U4	Lipoprotein	-	-	-
135	V8KTK9	Glutamate mutase	-	-	-
136	V8K149	Formate hydrogenlyase maturation protein HycH	-	-	-
137	V8KFV6	DNA damage-indicible protein DnaD	-	-	-
138	V8K463	Deubiquitinase	-	-	-
139	V8KT14	Acyl-CoA synthetase	-	-	-
140	V8KCI2	Aankyrin	-	-	-
141	V8KH92	4-hydroxybenzoate decarboxylase	-	-	-
142	V8KVD8	3-methylaspartate ammonia-lyase	-	-	-

'-' : not defined
